# Comparison of Immunogenicity and Safety between a Single Dose and One Booster Trivalent Inactivated Influenza Vaccination in Patients with Chronic Kidney Disease: A 20-Week, Open-Label Trial

**DOI:** 10.3390/vaccines9030192

**Published:** 2021-02-25

**Authors:** Yu-Tzu Chang, Tsai-Chieh Ling, Ya-Yun Cheng, Chien-Yao Sun, Jia-Ling Wu, Ching Hui Tai, Jen-Ren Wang, Junne-Ming Sung

**Affiliations:** 1 Department of Internal Medicine, National Cheng Kung University Hospital, College of Medicine, National Cheng Kung University, Tainan 704, Taiwan; kangxiemperor@gmail.com (Y.-T.C.); perfect5751@hotmail.com (T.-C.L.); chienyaosun@gmail.com (C.-Y.S.); balletwu72@gmail.com (J.-L.W.); 2 Department of Environmental Health, T.H Chan School of Public Health, Harvard University, Boston, MA 02115, USA; b507092063@tmu.edu.tw; 3 Department of Medical Laboratory Science and Biotechnology, College of Medicine, National Cheng Kung University, Tainan 704, Taiwan; e480216@hotmail.com (C.H.T.); jrwang@mail.ncku.edu.tw (J.-R.W.); 4 National Institute of Infectious Diseases and Vaccinology, National Health Research Institutes, Tainan 704, Taiwan

**Keywords:** non-dialysis-dependent chronic kidney disease, influenza virus, vaccine, booster dosage, immune response

## Abstract

Background: Non-dialysis-dependent chronic kidney disease (CKD-ND) patients are recommended to receive a one-dose influenza vaccination annually. However, studies investigating vaccine efficacy in the CKD-ND population are still lacking. In this study, we aimed to evaluate vaccine efficacy between the one-dose and two-dose regimen and among patients with different stages of CKD throughout a 20-week follow-up period. Methods: We conducted a single-center, non-randomized, open-label, controlled trial among patients with all stages of CKD-ND. Subjects were classified as unvaccinated, one-dose, and two-dose groups (4 weeks apart) after enrollment. Serial changes in immunological parameters (0, 4, 8, and 20 weeks after enrollment), including seroprotection, geometric mean titer (GMT), GMT fold-increase, seroconversion, and seroresponse, were applied to evaluate vaccine efficacy. Results: There were 43, 84, and 71 patients in the unvaccinated, one-dose, and two-dose vaccination groups, respectively. At 4–8 weeks after vaccination, seroprotection rates in the one- and two-dose group for H1N1, H3N2, and B ranged from 82.6–95.8%, 97.4–100%, and 73.9–100%, respectively. The concomitant seroconversion and GMT fold-increases nearly met the suggested criteria for vaccine efficacy for the elderly population. Although the seroprotection rates for all of the groups were adequate, the seroconversion and GMT fold-increase at 20 weeks after vaccination did not meet the criteria for vaccine efficacy. The two-dose regimen had a higher probability of achieving seroprotection for B strains (Odds ratio: 3.5, 95% confidence interval (1.30–9.40)). No significant differences in vaccine efficacy were found between early (stage 1–3) and late (stage 4–5) stage CKD. Conclusions: The standard one-dose vaccination can elicit sufficient protective antibodies. The two-dose regimen induced a better immune response when the baseline serum antibody titer was low. Monitoring change in antibody titers for a longer duration is warranted to further determine the current vaccine strategy in CKD-ND population.

## 1. Introduction

Infection is one of the leading causes of mortalities in the chronic kidney disease (CKD) population [1]. It is attributable to a dysfunction of the immune system in CKD patients, including both the innate and adaptive immune systems [2]. Multiple factors related to CKD are known to contribute to immune dysfunction, including accumulated uremic toxins, increased oxidative stress, endothelial dysfunction, a pro-inflammatory state, metabolic disturbances (mineral and bone disorders, erythropoietin deficiency, and renin-angiotensin-aldosterone system activation) and intestinal dysbiosis [2,3,4]. In addition, CKD patients are usually elderly or comorbid with many chronic illnesses, which will make them more prone to infection due to frequent medical visits.

Since the risk of influenza-associated hospitalization and mortality is high in CKD patients [5,6], international guidelines, including Kidney Disease Improving Global Outcomes (KDIGO), and the Advisory Committee on Immunization Practices (ACIP) from the Centers for Disease Control and Prevention (CDC), recommend an annual one-dose influenza vaccination for primary prevention [7,8,9]. However, several studies have suggested that vaccine immunogenicity becomes worse with decreases in the glomerular filtration rate [10,11,12,13]. Furthermore, the approach of one-standard-dose influenza vaccination does not induce sustained protective antibodies till the end of the flu season [14]. Multiple researchers have been working on strategies to improve influenza vaccine immunogenicity [15], including managements as booster vaccinations [14,16,17,18], high dose vaccines [19], and adjuvanted vaccines [20,21,22,23,24]. However, there is no definite consensus on optimal dosage and administration of influenza vaccines in the CKD population, and studies targeting the non-dialysis-dependent CKD (CKD-ND) population are especially scarce [25]. In this study, we compared the efficacy of one-dose and two-dose regimens and explored immunogenicity among patients at different stages of CKD after influenza vaccination over a 20-week follow-up period.

## 2. Materials and Methods

### 2.1. Study Design and Group Allocation of the Enrolled Subjects

During the 2013–2014 influenza season, we conducted this open-label, controlled trial to investigate the immunogenicity and safety of the different dosages (one-dose or two-dose) of the trivalent inactivated seasonal influenza vaccine in CKD-ND patients. This study was conducted after the approval of the Institutional Review Board (IRB) of National Cheng Kung University Hospital (IRB number: A-BR-101-139) and was then registered in Clinicaltrials.gov (NCT02105519). All subjects were recruited from National Cheng Kung University Hospital, a tertiary medical center in southern Taiwan. Eligibility requirements included CKD-ND men and non-pregnant women aged ≥ 18 years who were naïve to the 2013–2014 influenza vaccination. Exclusion criteria included hypersensitivity to eggs or egg protein, a personal or family history of Guillain–Barré Syndrome, acute febrile illness within the past week, upper respiratory illness within the prior 3 days, history of immunodeficiency, or taking any immunosuppressive agent and being hospitalized or receiving any blood products in the previous 3 months.

To simultaneously evaluate the immunogenicity of the influenza vaccination and control the potential confounding effect of a clinical influenza infection, we divided the enrolled subjects into 3 groups. Patients who refused to receive the vaccination were classified into the unvaccinated group, which made it possible to construct a surveillance system by which to monitor local influenza virus activity. Patients in the one-dose and two-dose group received one dose of vaccination at enrollment, and those in the two-dose group received one booster dose 4 weeks after the first vaccination. Since we intended to enroll more than 50 subjects in either the one- or two-dose group, as suggested by European guidelines for influenza trials [26], a power calculation was not performed. Informed written consent was obtained from all study participants. To elucidate dynamic changes in the immunogenicity profile of the CKD-ND patients, serum samples were collected at a serial time points, week 0 (before the vaccination) and at 4, 8, and 20 weeks after enrollment.

### 2.2. Vaccine Formula and Monitoring of Adverse Effects Related to Vaccination

The vaccine used in this study was the trivalent, non-adjuvanted, inactivated split-virion vaccine (AdimFlu-S, Adimmune Corporation, Taichung, Taiwan), which contained 15 μg of hemagglutinin (HA) antigen for each strain. It was produced in embryonated chicken eggs, was inactivated with formaldehyde, and was diluted with a phosphate buffered solution to formulate a final solution containing the required amount of HA from the virus strain. The virus strains contained in the vaccine, including A/California/7/2009 A pdm09 (Reassortant NYMC X-179A)(H1N1), A/Texas/50/2012 (H3N2), and B/Massachusetts/2/2012 (Reassortant NYMC BX-51B), followed the recommendation of the World Health Organization (WHO) for the 2013–2014 influenza season in the Northern Hemisphere. All subjects in the vaccination group received one dose of vaccine at each time point by intramuscular injection into the deltoid region. Safety data consisting of serious and non-serious adverse events were monitored throughout the entire study period, and reactogenicity events, including systemic and local adverse effects, were recorded once daily on a diary card for 7 consecutive days after vaccination.

### 2.3. Parameters of Immunogenicity Based on Hemagglutination-Inhibition (HI) Assays

All serum samples were frozen at −80 °C after sampling until analysis. To avoid any potential bias, the serum samples were masked with serial number tags before being sent for analysis. The HI assays were measured by strictly following the standard protocol suggested by the WHO [5]. In brief, we first treated all serum samples with receptor-destroying enzyme and 0.75% human red blood cells to inactivate non-specific inhibitors of hemagglutination and to remove non-specific agglutinins, respectively. Then, the serum samples were diluted with a serial 2-fold dilution in the 96-well microtiter plates with “V” shape bottom at the initial dilution level of 1:10. All diluted serum samples were mixed with 4 viral hemagglutinin units of influenza virus antigen for 15 min. Human type O RBC suspension in phosphate buffered saline (0.75%) was then added, and HI titers were read after 30–60 min incubation at room temperature. The HI titers were recorded as the reciprocal of the highest serum dilution test that completely inhibited hemagglutination, where antibody titers of less than 1:10 were recorded as 1:5 when analyzed. All samples were first measured in duplication, and a third examination was performed if the results were not consistent with each other. Vaccine efficacy was assessed by four immunogenicity parameters, which were suggested by the international guidelines [27,28]. The parameters are defined as follows: (a) seroprotection rate (HI antibody titer ≥ 1:40), (b) seroconversion rate (≥4-fold HI titer with the HI titer ≥ 1:40 after vaccination), (c) seroresponse rate (≥4-fold increase in the HI titer after vaccination), (d) geometric mean titer (GMT), and GMT fold-increase.

### 2.4. Statistical Analysis

Comparisons of between-group differences were performed using a one-way analysis of variance or the Kruskal–Wallis test for the continuous variables, and the Chi-Square test or the Fisher’s exact test were used for the categorical variables where appropriate. The 95% confidence intervals (CIs) for the seroprotection, seroresponse, and seroconversion rates were estimated using the Clopper–Pearson method. GMTs and the associated 95% CIs were calculated by taking the exponential of the log of the means and their 95% CIs. To clarify whether the two-dose regimen or differences in CKD stage would affect the vaccine efficacy (seroprotection and seroresponse), we constructed multivariate logistic regression models with generalized estimating equations (GEEs) based on exchangeable working correlation matrices to simultaneously control for potential confounders. Differences in the seroprotection and GMT of the same group at various time points were also assessed using the GEE models due to the nature of the repeated measurements. A two-sided *p*-value less than 0.05 was considered to be statistically significant. All statistical analyses were performed with SAS version 9.4 (SAS Institute, Cary, NC, USA), and graphs were depicted using Excel software (Microsoft, Washington, DC, USA).

## 3. Results

### 3.1. Baseline Characteristics of the Enrolled Patients

**Figure 1:**
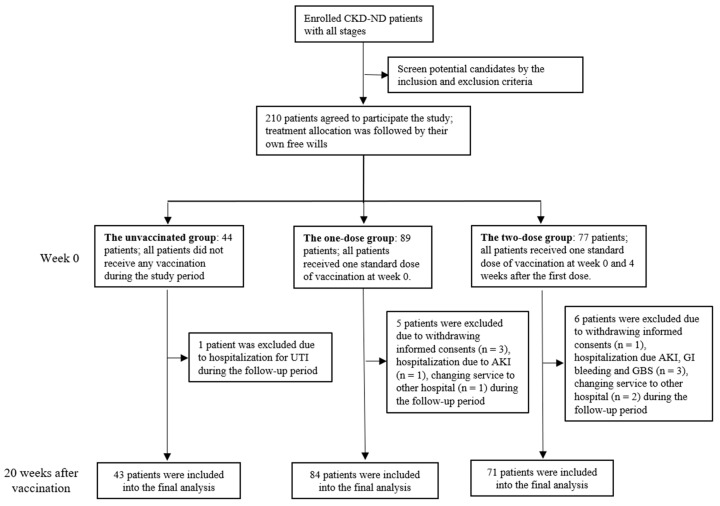
Flow chart of the selection of the study population. Abbreviation: CKD-ND: non-dialysis-dependent chronic kidney disease, UTI: urinary tract infection, AKI: acute kidney injury, GBS: Guillain–Barré syndrome.

Initially, 210 patients at different stages of CKD were enrolled in the study. During the 20-week study period, 12 patients dropped out of the study due to withdrawal of the informed consents (n = 4), changing the service to other hospitals (n = 3), hospitalization due to acute kidney injury, urinary tract infection, gastrointestinal bleeding, and suspicion of Guillain–Barré syndrome (GBS) (n = 5). Finally, there were 43, 84, and 71 patients in the unvaccinated, one-dose, and two-dose vaccination groups, respectively (Figure 1). Although not randomized, there were no significant between-group differences in age, gender, comorbidities, hematocrit, biochemical profile, or nutrition markers (serum phosphate, albumin, total cholesterol, and triglyceride) (Table 1).

### 3.2. Change in Immunogenicity Parameters Based on HI Assays before and after Vaccination

**Figure 2:**
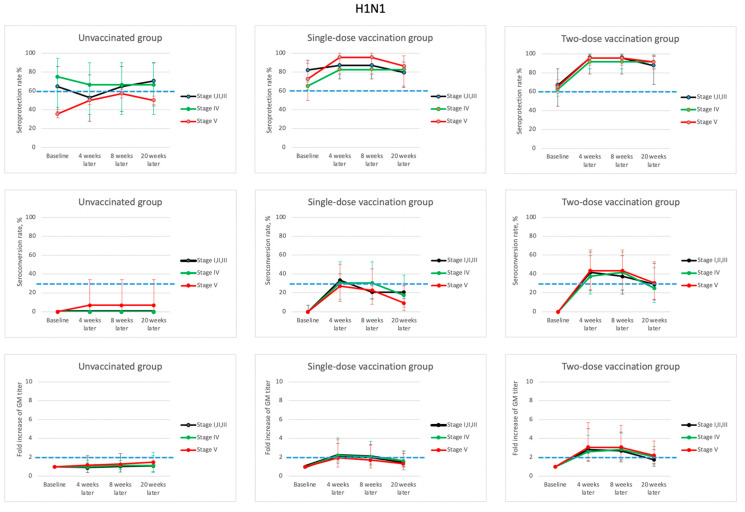
Dynamic changes of hemagglutination-inhibition (HI) antibody response (seroprotection rate, seroconversion rate, and fold of geometric mean (GM) titer) with their corresponding 95% confidence interval for influenza A (H1N1) during the 5-month study period for patients in various stages of chronic kidney disease (CKD) and different vaccination dosages. (Blue dash lines indicate the suggested threshold values of vaccine efficacy for patients > 60 years old: seroprotection rate > 60%; seroconversion rate > 30%, and fold increase of GM titer > 2).

**Figure 3:**
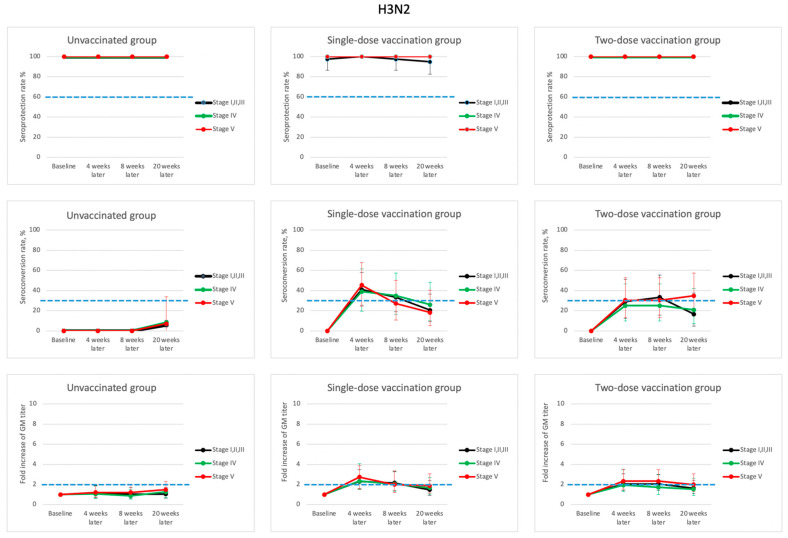
Dynamic changes of hemagglutination-inhibition (HI) antibody response (seroprotection rate, seroconversion rate and fold of geometric mean (GM) titer) with their corresponding 95% confidence interval for influenza A (H3N2) during the 5-month study period for patients in various stages of chronic kidney disease (CKD) and different vaccination dosages. (Blue dash lines indicate the suggested threshold values of vaccine efficacy for patients > 60 years old: seroprotection rate > 60%; seroconversion rate > 30%, and fold increase of GM titer > 2).

**Figure 4:**
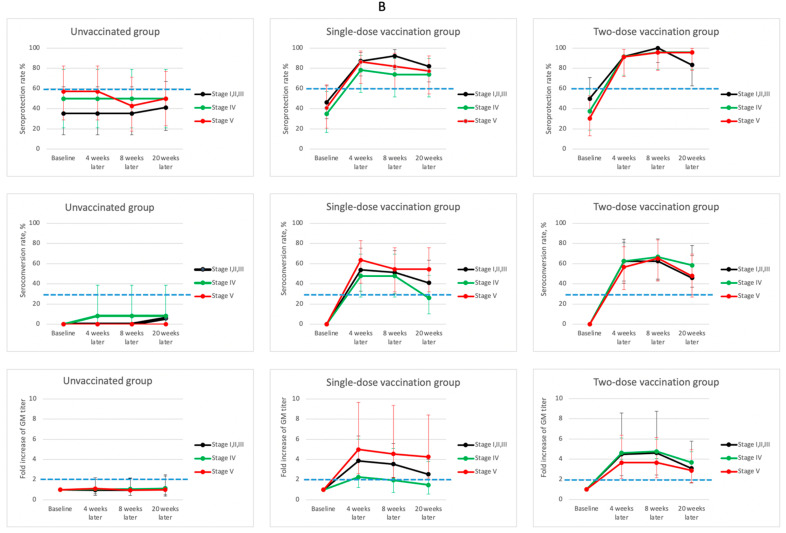
Dynamic changes of hemagglutination-inhibition (HI) antibody response (seroprotection rate, seroconversion rate, and fold of geometric mean (GM) titer) with their corresponding 95% confidence interval for influenza B during the 5-month study period for patients in various stages of chronic kidney disease (CKD) and different vaccination dosages. (Blue dash lines indicate the suggested threshold values of vaccine efficacy for patients > 60 years old: seroprotection rate > 60%; seroconversion rate > 30%, and fold increase of GM titer > 2).

**Figure 5:**
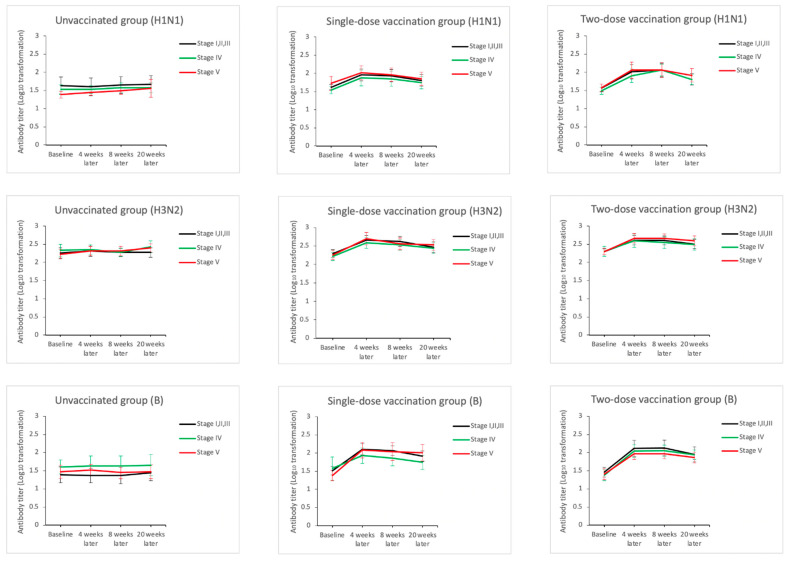
Dynamic changes in the log_10_ transformed hemagglutination-inhibition titers for H1N1, H3N2, and B influenza virus strains after stratification based on vaccination dosage (the unvaccinated, one-dose, and two-dose groups) and chronic kidney disease stages (stages 1–3, 4, and 5).

Figure 2, Figure 3, Figure 4 and Figure 5 and Supplementary Tables S1–S3 reveal the dynamic changes in immunogenicity before and after vaccination during the study period. For the H1N1 virus strain (Figure 2 and Supplementary Table S1), the baseline seroprotection rates of all unvaccinated and vaccinated groups at various stages of CKD ranged from 35.7~82.1%, while most of them were above 62.5%. Among all patients in the one- or two-dose groups, the seroprotection rates increased to 82.6~95.7% at 4 weeks after enrollment. In addition, the GMT fold-increases and the seroconversion rates were mostly over 2.2 and 30.4%, which met the minimum criteria for influenza vaccine efficacy for the elderly population, as suggested by the international guidelines (seroprotection > 60%, GMT fold-increase > 2.0, and seroconversion > 30%) [27,28]. Among the patients in the two-dose group, the booster dose did not induce significantly higher seroprotection, seroconversion, or GMT fold-increases 8 weeks after enrollment as compared to those at 4 weeks after enrollment.

For the H3N2 virus strain (Figure 3 and Supplementary Table S2), the baseline seroprotection rates of all the unvaccinated and one- and two-dose groups were nearly 100%. Therefore, the seroprotection rates remained at nearly 100% after the one or two dose regimens 4–20 weeks after enrollment. Considering other immunological parameters 4 weeks after enrollment, most of the GMT fold-increases and seroconversion values in both the one- and two-dose groups were above 2.0 or 30%, respectively. The booster dose did not elicit any significant improvements in these immunological parameters as compared to the one-dose group 8 weeks after vaccination.

For the B virus strain (Figure 4 and Supplementary Table S3), the baseline seroprotection rate of each group, ranging 30.4–57.1%, was the lowest among the three virus strains, with the exception of the subgroup of unvaccinated CKD stage 5 patients. After one dose of vaccination, significant elevations in seroprotection, seroconversion, and GMT fold-increases were observed 4 weeks after enrollment, and all of these values were higher than 78.3%, 47.8%, and 2.3, respectively. One booster regimen further increased seroprotection rates from 91.3–91.7% to 95.7–100%, across the CKD groups 8 weeks after enrollment. However, no incremental increases in the GMT fold-increase and seroconversion were found 8 weeks after enrollment.

When considering the dynamic changes in the immunological parameters for all three virus strains 8–20 weeks after enrollment, the seroprotection, seroconversion, and GMT values, and GMT fold-increases in both the one-dose and two-dose groups declined gradually over time, especially seroconversion and GMT fold-increase values for influenza A. Among the unvaccinated group, the changes in all immunological parameters remained relatively stable during the entire study period, with the exception of the HI titer levels for H3N2 in the unvaccinated CKD stage 5 subgroup. This suggested that there was no widespread influenza infection within the study population during the study period and that the antibody responses in the vaccinated groups were induced by the vaccines rather than by infection.

### 3.3. Determining Factors for Seroprotection and Seroresponse among the One- and Two-Dose Vaccination Groups

Among all of the vaccinated patients, the study results suggested that the two-dose vaccination strategy elicited a significantly higher chance to achieve seroprotection against the influenza B virus strain, but not the H1N1 and H3N2 strains (Table 2). The comparison of the vaccine efficacy for the different stages of CKD revealed that neither CKD stage 4 nor 5 was associated with lower seroprotection or seroresponse for any of the three virus strains after vaccination. Furthermore, seroprotection before vaccination was the predictor for achieving seroprotection after immunization. Aging was associated with lower seroprotection and seroresponse against H1N1 and B strains, but not against the H3N2 strain.

### 3.4. Local and Systemic Adverse Events Related to Vaccination during the Study Period

Table 3 reveals the local and systemic events occurring within one week after vaccination. In general, most of the systemic or local adverse effects were mild. Some of the symptoms, including nasal congestion, cough, muscle aches, and malaise, were over 10%. Only 3.37% of the participants reported having moderate to severe adverse effects (malaise). No participants were hospitalized due to respiratory tract diseases. However, one patient was admitted to another hospital for suspicion of GBS after receiving the two–dose vaccination regimen.

## 4. Discussion

According to the ACIP, an annual one dose of influenza vaccination is recommended for CKD patients [9]. However, to the best of our knowledge, there have been no studies investigating dynamic changes in the immune response to influenza vaccination in CKD-ND patients, which indicates that the evidence supporting the ACIP suggestion is not solid. In this study, our results demonstrated that one standard dose of seasonal influenza vaccine could elicit adequate post-vaccination seroprotection rates for three vaccine virus strains during a 20-week follow-up period (all >70%). When assessing vaccine immunogenicity by other parameters 4–8 weeks after vaccination, most of the post-vaccination seroconversion values and GMT fold-increases in all of the CKD subgroups met the criteria for vaccine efficacy set for the elderly population. In addition, the one booster dose regimen had higher probability of achieving seroprotection against the B strain(Odds ratio:3.5, 95% CI (1.30–9.40)). There were no significant between-group differences in vaccine efficacy for the mild–moderate (stage 1–3) and severe (stage 4–5) CKD subjects. Finally, the decline in seroconversion values and GMT fold-increases 20 weeks after vaccination raised concern as to whether the vaccine-induced protective effect could be sustained for one year. Therefore, our study results expanded the existing body of evidence suggesting that the current influenza vaccination strategy is applicable to the CKD-ND population, and one booster dose might be considered if the baseline seroprotective antibody titer is predicted to be low, e.g., when patients are vaccine-naïve or when the suggested strain has not been included in previous years.

In a real-world setting, a certain fraction of the study population would have high pre-vaccination HI titers because these patients could have been exposed to similar viral antigens before, either from a circulating virus or vaccine strains. Although a high post-vaccination HI titer (seroprotection rate) is one of the criteria for evaluating vaccine efficacy, especially from the perspective of public health, a high post-vaccination HI titer can also be a result of a high pre-vaccination HI titer, which will inevitably lead to overestimation of vaccine efficacy [29]. In contrast, the application of GMT fold-increases and seroconversion rates will lead to underestimation of vaccine efficacy if there is a high pre-vaccination HI titer [29]. To reduce such potential estimation bias, we chose an alternative method by constructing multivariate logistic regression models with generalized estimating equations to evaluate vaccine efficacy. The advantage of this approach was that it could facilitate simultaneous adjustment of pre-vaccination HI titer levels and baseline characteristics as well as changes in serological parameters at different time points.

The current immunization protocol should elicit and maintain enough high protective antibody levels to span the entire flu season. This is especially important for immunocompromised patients because the rapid decline in protective antibodies may have patients re-exposed to risk of influenza infection, which is similar to the phenomenon in patients after the COVID-19 virus infection [30,31]. Nevertheless, few studies have addressed this issue, and the results have been inconsistent [32,33,34,35]. A review of influenza vaccinations in an elderly population suggested the induced antibody response could be maintained for 4–6 months [32]. The results from solid organ transplant recipients indicated the standard one-dose influenza vaccination could not induce optimal or sustainable immune responses [33,34,35]. Therefore, multiple strategies have been proposed to improve immunogenicity, including adjuvanted vaccines and high-dose vaccines. Since kidney transplant recipients can be viewed as a specific form of CKD, evidence from this population might be directly applicable in the CKD-ND population. The adjuvanted vaccine was not shown to elicit better immunogenicity than a standard vaccine [20]. In contrast, higher dose influenza vaccination strategies seem to hold promise in kidney transplant recipients. In two randomized controlled trials, which enrolled 79.7% and 39% kidney transplant recipients, respectively, either the double dose (30 μg for each strain) or high dose (60 μg for each strain) regimen exhibited significantly better immunological parameters, including seroprotection, post-vaccination GMT and seroconversion as compared with the standard one-dose regimen [34,36]. In our study, a decline in protective antibodies was observed in both the one-dose and two-dose groups. The application of the double-dose or high-dose approach could be another potential strategy to enhance immunogenicity in the CKD-ND population.

The study results revealed that the booster dose strategy could improve immunogenicity against the influenza B strain but not the A strain (H1N1 and H3N2). This might have been due to the relatively lower HI titer level for the B strain at baseline in our study population. In 2013–2014, the recommended composition of influenza vaccine for H1N1 and H3N2 following the WHO was adopted for 5 and 2 consecutive years, respectively. Furthermore, the circulating influenza virus strain during the influenza season 2012–2013 in Taiwan was the same as the 2012–2013 WHO recommended H3N2 vaccine strain, A/Victoria/361/2011-like virus [37,38]. It is reasonable to speculate that many individuals in Taiwan might have been exposed to either the circulating or vaccine strains, which evoked the production of protective antibodies against the A/Victoria/361/2011-like virus before the influenza season 2013–2014. On the other hand, this was the first time the B strain (B/Massachusetts/2/2012-like virus) was recommended. It might partly explain the high pre-vaccination seroprotection rate for H3N2 in our study, which was conducted during influenza season 2013–14. This speculation is further supported by the findings that baseline seroprotection rates for H3N2 (97.0%, 95% confidence interval [CI]: 91.5, 99.4)) were also high in one clinical trial conducted in Taiwan among children and adolescents during 2013–2014 [39]. Previous studies investigating the immunogenicity between one dose and a booster dose also indicated the superiority of the booster strategy when the baseline protective antibody level is low, where the effects are attenuated if the baseline antibody level is high [16,35,40]. These results correspond to the findings of the present study and suggest that a lower level of baseline protective antibodies may accentuate the benefit of the booster vaccine.

GBS has become one of the major vaccine safety concerns since the influenza A/New Jersey/1976 vaccine was found to be associated with an eight-fold increased risk of GBS [41]. However, the following series of studies demonstrated that an increased risk of GBS attributable to vaccination is minimal, ranging from 1 to 3 additional cases of GBS per million doses administered [41]. Actually, influenza infection could induce the development of GBS. The incidence of GBS admission is 17.2 per million influenza encounters, which is significantly higher than those receiving vaccinations (1.03 per million vaccinations) [42]. Since the risk of GBS following influenza infection is far greater than the risk following influenza vaccination, influenza vaccination may play a protective role in reducing the risk of GBS as a trade-off. Vellozz et al. demonstrated that 2009 pH1N1-vaccinated individuals had lower cumulative GBS risk than those without vaccination (6.6 vs. 9.2 cases per million persons, *p* = 0.012) [43]. Therefore, we suggest that the minimal increase of GBS risk following influenza vaccination might not preclude a decision to receive a vaccination.

Several limitations of this study should be addressed. First, our treatment allocation was not randomized. Potential confounding by indication or unmeasurable covariates could not be minimized, and thus, causation could not be definitely established. In addition, the difference of baseline HI titers between groups made it difficult to directly compare the difference of immunogenicity. Therefore, we applied the GEE models to compare the difference of immunogenicity between different time points of the same group or to control for potential confounders, including seroprotection at baseline, while comparing the group difference. Furthermore, the application of GEE models could avoid the error of alpha inflation while conducting multiple comparisons between groups. Through this approach, we could reduce residual confounding to the greatest degree possible. Second, we stratified the participants by the pre-defined vaccination schedule and CKD stages. The use of this process might have made it possible to clearly present the differences in the immunological profiles between the various groups. Although we had a total sample size comparable to that of other influenza vaccine trials, the stratification strategy resulted in a small sample size in each stratum and limited the power of the study. It is also a major concern that we didn’t further perform age stratification to meet the criteria for vaccine efficacy suggested by the guidelines. Nevertheless, the results derived from the GEE models also led to the same conclusion and suggested that the results were robust. Third, we only evaluated vaccine efficacy using immunological profiles. Whether the humoral response can be directly translated into clinical outcomes, such as hospitalization for respiratory tract infection or mortality, is uncertain. In addition, cell-mediated immunity indeed plays an important role in both protecting the host from influenza infection and developing long-term immunological memory. The emerging evidence reveals that evaluation of vaccine efficacy by cell-mediated immune response might be an alternative approach and the correlation between clinical protection and parameters of cell-mediated immune response needs to be established [44]. It can provide a more comprehensive view if the influenza vaccine efficacy can be evaluated from all the clinical outcomes, humoral response, and cell-mediated immune response. Thus, caution should be taken when interpreting the study results. Fourth, we did not schedule patients in the unvaccinated group to record the discomforts listed in the Table 3. Therefore, it is unclear whether the systemic or local adverse effects reported by these patients within one week after influenza vaccination could be entirely attributable to vaccination.

In conclusion, the application of non-adjuvanted, trivalent, inactivated influenza vaccines in the CKD-ND population during the 2013–2014 influenza season elicited an adequate post-vaccination immune response for all three vaccine virus strains. The decline in the HI titers at 20 weeks after vaccination might indicate that CKD-ND patients could be re-exposed to the risk of developing influenza-associated complications after this time point. Furthermore, the two-dose regimen had the potential to induce significantly higher protective antibodies if the baseline antibody levels were low. No differences in the immunologic response were noticed among the various CKD groups. Further studies are required to define the optimal dosage and schedule of influenza vaccines for improvements in long-term immunogenicity in this population.

## Figures and Tables

**Table 1:** Baseline characteristics of the enrolled unvaccinated, one-, or two-dose influenza vaccination groups.

	All (n = 198)	The Unvaccinated Group (n = 43)	The One-Dose Vaccination Group (n = 84)	The Two-Dose Vaccination Group (n = 71)	*p*-Value ^b^
Age (years)	64.14 ± 11.76	65.35 ± 13.19	65.67 ± 11.67	61.61 ± 10.65	0.075
Men/Women (n)	117/81	21/22	50/34	46/25	0.243
Dry weight (Kgw)	65.11 ± 12.11	63.08 ± 12.88	65.50 ± 11.92	66.06 ± 11.81	0.444
Underlying disease					
Hypertension (n, %)	125 (63.13)	30 (69.77)	50 (59.52)	45 (63.38)	0.526
Diabetes (n, %)	83 (41.92)	23 (53.49)	31 (36.90)	29 (40.85)	0.195
Liver disease (n, %)	34 (17.17)	8 (18.60)	15 (17.86)	11 (15.49)	0.8911
Cardiovascular disease (n, %)	40 (20.20)	9 (20.93)	18 (21.43)	13 (18.31)	0.8824
Hematology					
White blood cell (10^3^/μL)	6.79 ± 1.86	6.27 ± 1.59	6.81 ± 1.92	7.07 ± 1.88	0.079
Hematocrit (%)	34.59 ± 5.73	33.96 ± 6.12	35.50 ± 5.63	33.90 ± 5.55	0.16
Platelet (10^3^/μL)	208.40 ± 59.91	190.81 ± 51.34	209.36 ± 66.40	217.92 ± 54.86	0.063
Biochemistry					
BUN (mg/dL)	42.61 ± 23.87	43.67 ± 27.28	38.89 ± 21.65	46.41 ± 23.86	0.142
Creatinine (mg/dL) ^a^	2.37 (2.35)	2.23 (3.18)	2.14 (1.85)	2.89 (2.67)	<0.0001
Glucose AC (mg/dL)	110.41 ± 37.10	102.63 ± 26.59	113.30 ± 35.42	111.70 ± 43.73	0.29
Calcium (mg/dL)	9.27 ± 0.54	9.22 ± 0.54	9.30 ± 0.52	9.26 ± 0.56	0.75
Phosphate (mg/dL)	3.92 ± 0.84	3.87 ± 0.99	3.84 ± 0.73	4.05 ± 0.86	0.272
Sodium (mmol/L)	141.61 ± 2.99	141.14 ± 3.62	141.36 ± 2.96	142.20 ± 2.52	0.111
Potassium (mmol/L)	4.36 ± 0.58	4.20 ± 0.66	4.37 ± 0.54	4.45 ± 0.56	0.093
Albumin (mg/dL)	4.26 ± 0.42	4.23 ± 0.41	4.27 ± 0.43	4.27 ± 0.43	0.808
Total-cholesterol (mg/dL) ^a^	153.00 (47.00)	158.00 (61.00)	154.00 (44.00)	152.00 (49.00)	0.095
Triglyceride (md/dL) ^a^	109.50 (71.00)	121.00 (66.00)	107.00 (77.00)	112.00 (74.00)	0.218
iPTH (pg/mL) ^a^	65.53 (64.40)	67.37 (90.92)	60.55 (58.69)	67.57 (63.33)	0.01

Values are expressed as mean ± standard deviation or median (interquartile range) ^a^ for continuous variables or number (percentage) for the categorical variables. Abbreviations: BUN: blood urea nitrogen; iPTH: intact parathyroid hormone. ^b^ The *p* values were calculated by comparing the differences between subjects receiving no, one, or two vaccinations using a one-way analysis of variance for the continuous variables or the chi-square test for the categorical.

**Table 2:** Determinants of seroprotection and seroresponse based on the multivariate logistic regression models with generalized estimating equations in patients with chronic kidney disease (CKD) receiving either one (n = 84) or two doses (n = 71) of influenza vaccination.

Variable	H1N1		H3N2		B	
	Seroprotection	Seroresponse	Seroprotection	Seroresponse	Seroprotection	Seroresponse
	OR (95% CI)	OR (95% CI)	OR (95% CI)	OR (95% CI)	OR (95% CI)	OR (95% CI)
Vaccination schedule (2 vs. 1 dose)	3.00 (0.94–9.63)	1.46 (0.76–2.81)	NA	0.71 (0.38–1.35)	3.50 (1.30–9.40)	1.17 (0.62–2.19)
CKD stage (Stage 4 vs. 1–3)	3.01 (0.76–11.98)	0.92 (0.39–2.17)	NA	0.76 (0.33–1.74)	0.61 (0.22–1.69)	0.70 (0.32–1.51)
CKD stage (Stage 5 vs. 1–3)	3.86 (0.59–25.41)	0.75 (0.30–1.85)	NA	0.74 (0.30–1.80)	0.56 (0.16–1.91)	0.62 (0.26–1.44)
Seroprotection before vaccination	56.17 (14.06–224.36)	0.88 (0.43–1.78)	NA	1.20 (0.61–2.33)	4.47 (1.75–11.44)	1.42 (0.72–2.81)
Age (year)	0.92 (0.87–0.97)	0.95 (0.92–0.98)	NA	0.99 (0.97–1.02)	0.96 (0.92–1.00)	0.94 (0.91–0.97)
Total–cholesterol (mg/dL)	1.00 (0.99–1.01)	1.00 (0.99–1.01)	NA	1.00 (1.00–1.01)	1.00 (0.99–1.01)	1.00 (0.99–1.01)
Hematocrit (%)	1.04 (0.91–1.19)	0.99 (0.92–1.06)	NA	0.95 (0.89–1.01)	0.95 (0.86–1.05)	0.92 (0.86–0.98)

Abbreviation: OR: Odds ratio, NA: not applicable; definition of seroprotection: hemagglutination-inhibition titers ≥ 1:40; seroresponse: ≥ 4–fold increase in antibody titer after vaccination.

**Table 3:** Systemic and local adverse effects within one week after influenza vaccination in patients with chronic kidney disease.

	The First Dose at Week 0		The Second Dose at 4 Weeks Later	
	Mild	Moderate to Severe	Mild	Moderate to Severe
**Systemic adverse effect**				
Fever (>38 °C)	1.87%	0.00%	0.75%	0.00%
Nasal congestion	10.86%	1.50%	1.12%	0.37%
Cough	11.61%	1.87%	1.87%	0.37%
Sore throat	4.49%	0.75%	1.12%	0.37%
Muscle aches	10.11%	2.25%	1.87%	0.00%
Headache	8.24%	1.50%	0.37%	0.00%
Nausea	3.75%	1.12%	0.37%	0.00%
Vomiting	1.87%	0.00%	0.75%	0.00%
Malaise	11.99%	3.37%	2.62%	0.00%
Eye redness	3.00%	0.37%	0.75%	0.00%
Chest tightness	3.75%	0.75%	0.75%	0.00%
Respiratory distress	2.62%	0.75%	0.37%	0.00%
Face edema	2.25%	0.37%	0.00%	0.00%
**Local adverse effect**				
Pain	9.36%	1.50%	0.75%	0.00%
Swelling	7.12%	1.12%	0.37%	0.00%
Redness	1.50%	0.37%	0.00%	0.00%
Ecchymosis	1.50%	0.37%	0.00%	0.00%
Decrease limb mobility	4.12%	0.00%	0.00%	0.00%

## Data Availability

Data can be requested by writing to the authors.

## Supplementary Materials

The following are available online at https://www.mdpi.com/2076-393X/9/3/192/s1, Table S1: Dynamic changes in hemagglutination-inhibition (HI) immunogenicity for influenza A (H1N1) during the 5-month study period for patients in various stages of chronic kidney disease (CKD); Table S2: Dynamic changes in hemagglutination-inhibition (HI) immunogenicity for influenza A (H3N2) in patients at various stages of chronic kidney disease (CKD) during the 5-month study period;. Table S3: Dynamic changes in hemagglutination-inhibition (HI) immunogenicity for influenza A (B) in patients at various stages of chronic kidney disease (CKD) during the 5-month study period.
