# Supplementary material for: Comparison of Immunogenicity and Safety between a Single Dose and One Booster Trivalent Inactivated Influenza Vaccination in Patients with Chronic Kidney Disease: A 20-Week, Open-Label Trial
Source: Vaccines (Basel). 2021 Feb 25;9(3):192. doi: 10.3390/vaccines9030192 (PMC7996510; doi:10.3390/vaccines9030192)
Supplement: Supplementary file 1 [file vaccines-09-00192-s001.pdf]

Supplementary Table 1. Dynamic changes in hemagglutination-inhibition (HI) immunogenicity for influenza A (H1N1) during the 5-month study period for patients in various stages of chronic kidney disease (CKD).

**CKD population (H1N1)**									
**Immunogenicity end point**	**The unvaccinated group (n=43)**			**The one-dose vaccination group (n=84)**			**The two-dose vaccination group (n=71)**		
	**Stage 1-3 (n=17)**	**Stage 4 (n=12)**	**Stage 5 (n=14)**	**Stage 1-3 (n=39)**	**Stage 4 (n=23)**	**Stage 5 (n=22)**	**Stage 1-3 (n=24)**	**Stage 4 (n=24)**	**Stage 5 (n=23)**
**Baseline (0 week)**									
Seroprotection rate % (95% CI)	64.7% (38.3-85.8)	75.0% (42.8-94.5)	35.7% (12.8-64.9)	82.1% (66.5-92.5)	65.2% (42.7-83.6)	72.7% (49.8-89.3)	66.7% (44.7-84.4)	62.5% (40.6-81.2)	65.2% (42.7-83.6)
GMT (95% CI)	43.4 (23.8-74.8)	33.6 (24.3-45.2)	24.4 (19.1-30.4)	41.4 (34.0-49.6)	34.4 (26.8-43.1)	53.1 (32.9-82.0)	36.7 (27.9-47.1)	30.8 (24.4-38.2)	37.7 (29.4-47.1)
**4 weeks later**									
Seroprotection rate % (95% CI)	52.9% (27.8-77.0)	66.7% (34.9-90.1)	50% (23.0-77.0)	87.2% (72.6-95.7)	82.6%[a] (61.2-95.0)	95.5%[a] (77.2-99.9)	95.8%[a] (78.9-99.9)	91.7%[a] (73.0-99.0)	95.7%[a] (78.1-99.9)
GMT (95% CI)	40.0 (21.6-69.9)	33.6 (25.7-42.9)	28.3 (23-34.1)	90.6[a] (59.4-132.9)	75.3[a] (42.8-125.7)	102.9[a] (62.6-161.7)	103.7[a] (62.4-164.5)	80.0[a] (50.5-121.4)	114.9[a] (64.9-192.9)
GMT fold-increase (95% CI)	0.9 (0.4-2.2)	1 (0.7-1.5)	1.2 (0.8-1.6)	2.2 (1.4-3.5)	2.2 (1.6-4.1)	1.9 (1-3.9)	2.8 (1.6-5)	2.6 (1.9-4.3)	3 (1.6-5.7)
Seroresponse rate % (95% CI)	0.0 (0.0-0.0)	0.0 (0.0-0.0)	7.1% (0.2-33.9)	33.3% (19.1-50.2)	30.4% (13.2-52.9)	27.3% (10.7-50.2)	41.7% (22.1-63.4)	37.5% (18.8-59.4)	43.5% (23.2-65.5)
Seroconversion rate % (95% CI)	0.0 (0.0-0.0)	0.0 (0.0-0.0)	7.1% (0.2-33.9)	33.3% (19.1-50.2)	30.4% (13.2-52.9)	27.3% (10.7-50.2)	41.7% (22.1-63.4)	37.5% (18.8-59.4)	43.5% (23.2-65.5)
**8 weeks later**									
Seroprotection rate %	64.7%	66.7%	57.1%	87.2%	82.6%[a]	95.5%[a]	95.8%[a]	91.7%[a]	95.7%[a]

(95% CI)	(38.3-85.8)	(34.9-90.1)	(28.9-82.3)	(72.6-95.7)	(61.2-95)	(77.2-99.9)	(78.9-99.9)	(73-99)	(78.1-99.9)
GMT<br>(95% CI)	45.2<br>(25.4-76.3)	37.8<br>(26.8-51.5)	31.2<br>(24.2-39.3)	85.9[a]<br>(56.3-125.9)	70.9[a]<br>(41.8-114.7)	90.7[a]<br>(56.8-138.8)	97.9[a]<br>(60-152.7)	87.2[a]<br>(57.5-127.3)	114.9[a]<br>(68.7-183.2)
GMT fold-increase<br>(95% CI)	1.0<br>(0.5-2.4)	1.1<br>(0.7-1.8)	1.3<br>(0.9-1.8)	2.1<br>(1.3-3.3)	2.1<br>(1.1-3.7)	1.7<br>(0.9-3.3)	2.7<br>(1.5-4.7)	2.8<br>(1.8-4.6)	3.0<br>(1.7-5.4)
Seroresponse rate %<br>(95% CI)	0.0<br>(0.0-0.0)	0.0<br>(0.0-0.0)	7.1%<br>(0.2-33.9)	20.5%<br>(9.3-36.5)	30.4%<br>(13.2-52.9)	22.7%<br>(7.8-45.4)	37.5%<br>(18.8-59.4)	41.7%<br>(22.1-63.4)	43.5%<br>(23.2-65.5)
Seroconversion rate %<br>(95% CI)	0.0<br>(0.0-0.0)	0.0<br>(0.0-0.0)	7.1%<br>(0.2-33.9)	20.5%<br>(9.3-36.5)	30.4%<br>(13.2-52.9)	22.7%<br>(7.8-45.4)	37.5%<br>(18.8-59.4)	41.7%<br>(22.1-63.4)	43.5%<br>(23.2-65.5)
**20 weeks later**									
Seroprotection rate %<br>(95% CI)	70.6%<br>(44.0-89.7)	66.7%<br>(34.9-90.1)	50%<br>(23.0-77.0)	79.5%<br>(63.5-90.7)	82.6%[a]<br>(61.2-95.0)	86.4%<br>(65.1-97.1)	87.5%[a]<br>(67.6-97.3)	91.7%[a]<br>(73.0-99.0)	91.3%[a]<br>(72.0-98.9)
GMT<br>(95% CI)	47.1<br>(26.0-80.8)	37.8<br>(26.8-51.5)	36.2<br>(19.4-63.9)	63.5 [a]<br>(42.7-91.1)	55.7[a]<br>(35.9-83.1)	70.5[a]<br>(44.6-106.9)	63.5[a]<br>(42.6-91.3)	63.5[a]<br>(45.0-86.8)	82.4[a]<br>(51.8-125.8)
GMT fold-increase<br>(95% CI)	1.1<br>(0.5-2.5)	1.1<br>(0.7-1.8)	1.5<br>(0.8-2.9)	1.5<br>(1.0-2.4)	1.6<br>(1-2.7)	1.3<br>(0.7-2.6)	1.7<br>(1.1-2.8)	2.1<br>(1.4-3.1)	2.2<br>(1.3-3.7)
Seroresponse rate %<br>(95% CI)	0.0<br>(0.0-0.0)	0.0<br>(0.0-0.0)	7.1%<br>(0.2-33.9)	20.5%<br>(9.3-36.5)	17.4%<br>(5.0-38.8)	9.1%<br>(1.1-29.2)	29.2%<br>(12.6-51.1)	25%<br>(9.8-46.7)	30.4%<br>(13.2-52.9)
Seroconversion rate %<br>(95% CI)	0.0<br>(0.0-0.0)	0.0<br>(0.0-0.0)	7.1%<br>(0.2-33.9)	20.5%<br>(9.3-36.5)	17.4%<br>(5.0-38.8)	9.1%<br>(1.1-29.2)	29.2%<br>(12.6-51.1)	25%<br>(9.8-46.7)	30.4%<br>(13.2-52.9)

CI: confidence interval; GMT: Geometric mean titer.

[a]*p*-value < 0.05 compared with the baseline value (week 0)

Supplementary Table 2. Dynamic changes in hemagglutination-inhibition (HI) immunogenicity for influenza A (H3N2) in patients at various stages of chronic kidney disease (CKD) during the 5-month study period.

CKD population (H3N2)									
**Immunogenicity end point**	**The unvaccinated group (n=43)**			**The one-dose vaccination group (n=84)**			**The two-dose vaccination group (n=71)**		
	**Stage 1-3 (n=17)**	**Stage 4 (n=12)**	**Stage 5 (n=14)**	**Stage 1-3 (n=39)**	**Stage 4 (n=23)**	**Stage 5 (n=22)**	**Stage 1-3 (n=24)**	**Stage 4 (n=24)**	**Stage 5 (n=23)**
**Baseline (0 week)**									
Seroprotection rate % (95% CI)	100%	100%	100%	97.4% (86.5-99.9)	100%	100%	100%	100%	100%
GMT (95% CI)	180.8 (125.1-252.6)	213.6 (138.9-315.7)	168.1 (120.7-227.1)	196.9 (151.0-250.5)	164.9 (123.4-214.5)	181.5 (131.6-242.9)	195.8 (158.6-237.2)	201.6 (142.7-275.7)	197.6 (155.6-245.4)
**4 weeks later**									
Seroprotection rate % (95% CI)	100%	100%	100%	100%	100%	100%	100%	100%	100%
GMT (95% CI)	204.3 (142.3-283.8)	226.3 (160.3-309.4)	204.9[a] (146.3-278.2)	454[a] (326.8-611.8)	383.4[a] (265.6-535)	497.4[a] (321.8-738.5)	403.2[a] (288.9-545.5)	391.7[a] (248.5-591.9)	459.4[a] (327.8-624.1)
GMT fold-increase (95% CI)	1.1 (0.7-1.9)	1.1 (0.6-1.8)	1.2 (0.8-2)	2.3 (1.5-3.5)	2.3 (1.6-3.7)	2.7 (1.6-4.7)	2.1 (1.4-3.1)	1.9 (1.3-3.4)	2.3 (1.5-3.5)
Seroresponse rate % (95% CI)	0.0 (0.0-0.0)	0.0 (0.0-0.0)	0.0 (0.0-0.0)	41% (25.6-57.9)	39.1% (19.7-61.5)	45.5% (24.4-67.8)	29.2% (12.6-51.1)	25% (9.8-46.7)	30.4% (13.2-52.9)
Seroconversion rate % (95% CI)	0.0 (0.0-0.0)	0.0 (0.0-0.0)	0.0 (0.0-0.0)	41% (25.6-57.9)	39.1% (19.7-61.5)	45.5% (24.4-67.8)	29.2% (12.6-51.1)	25% (9.8-46.7)	30.4% (13.2-52.9)
**8 weeks later**									
Seroprotection rate %	100%	100%	100%	97.4%	100%	100%	100%	100%	100%

(95% CI)				(86.5-99.9)					
GMT<br>(95% CI)	188.3<br>(139.3-247.7)	190.3<br>(145.4-242.8)	204.9<br>(146.3-278.2)	422.8[a]<br>(301.4-574.8)	339.9[a]<br>(239.2-467.4)	363[a]<br>(242.9-522.7)	403.2[a]<br>(296.4-533.1)	349[a]<br>(232.5-504.5)	459.4[a]<br>(332.2-616.6)
GMT fold-increase<br>(95% CI)	1.0<br>(0.6-1.7)	0.9<br>(0.5-1.5)	1.2<br>(0.8-2)	2.1<br>(1.4-3.3)	2.1<br>(1.3-3.2)	2.0<br>(1.2-3.3)	2.1<br>(1.4-3)	1.7<br>(1-2.9)	2.3<br>(1.6-3.5)
Seroresponse rate %<br>(95% CI)	0.0<br>(0.0-0.0)	0.0<br>(0.0-0.0)	0.0<br>(0.0-0.0)	33.3%<br>(19.1-50.2)	34.8%<br>(16.4-57.3)	27.3%<br>(10.7-50.2)	33.3%<br>(15.6-55.3)	25%<br>(9.8-46.7)	30.4%<br>(13.2-52.9)
Seroconversion rate %<br>(95% CI)	0.0<br>(0.0-0.0)	0.0<br>(0.0-0.0)	0.0<br>(0.0-0.0)	33.3%<br>(19.1-50.2)	34.8%<br>(16.4-57.3)	27.3%<br>(10.7-50.2)	33.3%<br>(15.6-55.3)	25%<br>(9.8-46.7)	30.4%<br>(13.2-52.9)
**20 weeks later**									
Seroprotection rate %<br>(95% CI)	100%	100%	100%	94.9%<br>(82.7-99.4)	100%	100%	100%	100%	100%
GMT<br>(95% CI)	188.3<br>(135.7-253.6)	269.1<br>(177.3-392.9)	249.8[a]<br>(185.4-327.4)	291.1[a]<br>(197.7-413.6)	275.2[a]<br>(191.3-383)	340.8[a]<br>(236-475.7)	320[a]<br>(231.2-429.7)	310.9[a]<br>(208.9-446)	395.2[a]<br>(279.9-540.4)
GMT fold-increase<br>(95% CI)	1.0<br>(0.6-1.7)	1.3<br>(0.7-2.3)	1.5<br>(1-2.3)	1.5<br>(0.9-2.4)	1.7<br>(1-2.7)	1.9<br>(1.2-3.1)	1.6<br>(1.1-2.4)	1.5<br>(0.9-2.6)	2.0<br>(1.3-3)
Seroresponse rate %<br>(95% CI)	5.9%<br>(0.1-28.7)	8.3%<br>(0.2-38.5)	7.1%<br>(0.2-33.9)	20.5%<br>(9.3-36.5)	26.1%<br>(10.2-48.4)	18.2%<br>(5.2-40.3)	16.7%<br>(4.7-37.4)	20.8%<br>(7.1-42.2)	34.8%<br>(16.4-57.3)
Seroconversion rate %<br>(95% CI)	5.9%<br>(0.1-28.7)	8.3%<br>(0.2-38.5)	7.1%<br>(0.2-33.9)	20.5%<br>(9.3-36.5)	26.1%<br>(10.2-48.4)	18.2%<br>(5.2-40.3)	16.7%<br>(4.7-37.4)	20.8%<br>(7.1-42.2)	34.8%<br>(16.4-57.3)

CI: confidence interval; GMT: Geometric mean titer.

[a]p-value < 0.05 compared with the baseline value (week 0)

Supplementary Table 3. Dynamic changes in hemagglutination-inhibition (HI) immunogenicity for influenza A (B) in patients at various stages of chronic kidney disease (CKD) during the 5-month study period.

**CKD population (B)**									
**Immunogenicity end point**	**The unvaccinated group (n=43)**			**The one-dose vaccination group (n=84)**			**The two-dose vaccination group (n=71)**		
	**Stage 1-3 (n=17)**	**Stage 4 (n=12)**	**Stage 5 (n=14)**	**Stage 1-3 (n=39)**	**Stage 4 (n=23)**	**Stage 5 (n=22)**	**Stage 1-3 (n=24)**	**Stage 4 (n=24)**	**Stage 5 (n=23)**
**Baseline (0 week)**									
Seroprotection rate % (95% CI)	35.3% (14.2-61.7)	50% (21.1-78.9)	57.1% (28.9-82.3)	46.2% (30.1-62.8)	34.8% (16.4-57.3)	40.9% (20.7-63.6)	50% (29.1-70.9)	37.5% (18.8-59.4)	30.4% (13.2-52.9)
GMT (95% CI)	24.5 (14.1-40.6)	40.0 (24.6-62.3)	29.7 (18.7-45.3)	32.9 (24.5-43)	37.7 (16.8-78.4)	24.2 (16.7-33.8)	29.1 (20.8-39.5)	23.8 (16.2-33.7)	25.5 (17.1-36.4)
**4 weeks later**									
Seroprotection rate % (95% CI)	35.3% (14.2-61.7)	50% (21.1-78.9)	57.1% (28.9-82.3)	87.2%[a] (72.6-95.7)	78.3%[a] (56.3-92.5)	86.4%[a] (65.1-97.1)	91.7% (73.0-99.0)	91.7%[a] (73.0-99.0)	91.3%[a] (72.0-98.9)
GMT (95% CI)	23.5 (14.2-37.3)	42.4 (21.0-80.0)	32.8 (22.0-47.1)	127.0[a] (85.3-182.2)	85.0 (48.7-140.7)	120.5[a] (69.8-197.7)	130.7[a] (75.3-215.6)	109.9[a] (71.4-162.7)	93.0[a] (63.1-132.2)
GMT fold-increase (95% CI)	1.0 (0.5-2.0)	1.1 (0.5-2.5)	1.1 (0.6-2.0)	3.9 (2.4-6.3)	2.3 (1.2-6.0)	5.0 (2.6-9.6)	4.5 (2.4-8.6)	4.6 (2.5-8.2)	3.7 (2.1-6.4)
Seroresponse rate % (95% CI)	0.0 (0.0-0.0)	8.3% (0.2-38.5)	0.0 (0.0-0.0)	53.8% (37.2-69.9)	47.8% (26.8-69.4)	63.6% (40.7-82.8)	62.5% (40.6-81.2)	62.5% (40.6-81.2)	56.5% (34.5-76.8)
Seroconversion rate % (95% CI)	0.0 (0.0-0.0)	8.3% (0.2-38.5)	0.0 (0.0-0.0)	53.8% (37.2-69.9)	47.8% (26.8-69.4)	63.6% (40.7-82.8)	62.5% (40.6-81.2)	62.5% (40.6-81.2)	56.5% (34.5-76.8)
**8 weeks later**									

Seroprotection rate % (95% CI)	35.3% (14.2-61.7)	50% (21.1-78.9)	42.9% (17.7-71.1)	92.3%[a] (79.1-98.4)	73.9%[a] (51.6-89.8)	81.8%[a] (59.7-94.8)	100.0%	95.8%[a] (78.9-99.9)	95.7%[a] (78.1-99.9)
GMT (95% CI)	23.5 (13.2-39.9)	42.4 (21.0-80.0)	28.3 (18.3-42.0)	116.2[a] (82.1-159.2)	73.1 (42.8-118.8)	109.6[a] (58.9-192.6)	134.5[a] (78.2-220.1)	113.1[a] (76.4-161.6)	93.0[a] (66.3-126.5)
GMT fold-increase (95% CI)	1.0 (0.4-2.1)	1.1 (0.5-2.5)	1.0 (0.5-1.8)	3.5 (2.2-5.6)	1.9 (0.7-5.1)	4.5 (2.2-9.4)	4.6 (2.4-8.8)	4.8 (2.7-8.2)	3.7 (2.2-6.1)
Seroresponse rate % (95% CI)	0.0 (0.0-0.0)	8.3% (0.2-38.5)	0.0 (0.0-0.0)	51.3% (34.8-67.6)	47.8% (26.8-69.4)	54.5% (32.2-75.6)	62.5% (40.6-81.2)	66.7% (44.7-84.4)	65.2% (42.7-83.6)
Seroconversion rate % (95% CI)	0.0 (0.0-0.0)	8.3% (0.2-38.5)	0.0 (0.0-0.0)	51.3% (34.8-67.6)	47.8% (26.8-69.4)	54.5% (32.2-75.6)	62.5% (40.6-81.2)	66.7% (44.7-84.4)	65.2% (42.7-83.6)
**20 weeks later**									
Seroprotection rate % (95% CI)	41.2% (18.4-67.1)	50% (21.1-78.9)	50% (23.0-77.0)	82.1%[a] (66.5-92.5)	73.9%[a] (51.6-89.8)	77.3%[a] (54.6-92.2)	83.3% (62.6-95.3)	95.8%[a] (78.9-99.9)	95.7%[a] (78.1-99.9)
GMT (95% CI)	27.7 (15.8-46.1)	44.9 (21.6-87.3)	29.7 (18.2-46.3)	82.9[a] (57.6-115.4)	55.7 (34.2-86.8)	102.9[a] (58.2-172.7)	89.8[a] (52.8-145.3)	87.2[a] (62.8-117.6)	73.1[a] (50.6-102.1)
GMT fold-increase (95% CI)	1.1 (0.5-2.5)	1.1 (0.5-2.7)	1.0 (0.5-2)	2.5 (1.6-4)	1.5 (0.6-3.8)	4.3 (2.2-8.4)	3.1 (1.6-5.8)	3.7 (2.2-6.1)	2.9 (1.7-4.9)
Seroresponse rate % (95% CI)	5.9% (0.1-28.7)	8.3% (0.2-38.5)	0.0 (0.0-0.0)	41% (25.6-57.9)	26.1% (10.2-48.4)	54.5% (32.2-75.6)	45.8% (25.6-67.2)	58.3% (36.6-77.9)	47.8% (26.8-69.4)
Seroconversion rate % (95% CI)	5.9% (0.1-28.7)	8.3% (0.2-38.5)	0.0 (0.0-0.0)	41% (25.6-57.9)	26.1% (10.2-48.4)	54.5% (32.2-75.6)	45.8% (25.6-67.2)	58.3% (36.6-77.9)	47.8% (26.8-69.4)

CI: confidence interval; GMT: Geometric mean titer.

[a]*p*-value < 0.05 compared with the baseline value (week 0)
